# Should I Rest or Should I Go Now? A Randomized Cross-Over Trial Comparing Fixed and Self-Selected Rest Durations in High-Intensity Interval Training Cycling Sessions

**DOI:** 10.1186/s40798-023-00601-8

**Published:** 2023-07-03

**Authors:** Eyal Colorni, Evyatar Ohayon, Julie N. Côté, Uri Obolski, Israel Halperin

**Affiliations:** 1 Department of Health Promotion, School of Public Health, Faculty of Medicine, Tel-Aviv University, Tel Aviv, Israel; 2 Sylvan Adams Sports Institute, Tel Aviv University, Tel Aviv, Israel; 3 Department of Kinesiology and Physical Education, McGill University, Montreal, QC Canada; 4 Occupational Biomechanics and Ergonomics Laboratory, Michael Feil and Ted Oberfeld/CRIR Research Centre, Jewish Rehabilitation Hospital, Laval, QC Canada; 5 Department of Epidemiology and Preventive Medicine, School of Public Health, Faculty of Medicine, Tel Aviv University, Tel Aviv, Israel; 6 Department of Environmental Studies, Porter School of the Environment and Earth Sciences, Faculty of Exact Sciences, Tel Aviv University, Tel Aviv, Israel

**Keywords:** Self-selected rest, HIIT, Autonomy-supportive coaching, Cyclists

## Abstract

**Background:**

In high-intensity interval training (HIIT), the rest durations between intervals are commonly prescribed using a fixed approach (e.g., 30 s between intervals). An alternative is the self-selected (SS) approach, in which trainees select their resting durations. Studies comparing the two approaches report mixed results. However, in these studies, trainees in the SS condition rested for as little or as long as they wished, leading to dissimilar total rest durations between conditions. Here, for the first time, we compare the two approaches while controlling for total rest duration.

**Methods:**

Twenty-four amateur adult male cyclists completed a familiarization session, followed by two counterbalanced cycling HIIT sessions. Each session was composed of nine, 30-s intervals, in which the goal was to accumulate as many watts as possible on an SRM ergometer. In the fixed condition, cyclists rested for 90 s between intervals. In the SS condition, cyclists had 720 s (i.e., 8  × 90 s) of rest to allocate in any way they wished. We measured and compared watts, heart rate, electromyography of the knee flexors and extensors, rating of perceived effort and fatigue, perception of autonomy and enjoyment. Additionally, a subsample of ten cyclists completed a retest of the SS condition.

**Results:**

With the exception of perception of autonomy, which was higher in the SS condition, outcomes were highly similar in both conditions. For example, the average aggregated differences were: 0.57 (95% CI − 8.94, 10.09) for watts; − 0.85 (95% CI − 2.89, 1.18) for heart rate; and 0.01 (95% CI − 0.29, 0.30) for rating of perceived effort (on a 0–10 scale). Additionally, the retest of the SS condition resulted in a similar rest allocation pattern across the intervals and in similar outcomes.

**Conclusion:**

Given the similarities in performance, physiological and psychological outcomes between the fixed and SS conditions, both can be equally utilized based on coaches’ and cyclists’ preferences and training goals.

**Supplementary Information:**

The online version contains supplementary material available at 10.1186/s40798-023-00601-8.

## Key points

- We compared the impact of fixed and self-selected rest durations during HIIT cycling sessions on performance, physiological and psychological outcomes, among male cyclists.
- In contrast to previous studies comparing fixed and self-selected rest durations, in the present study we matched total rest durations between the two conditions.
- We observed highly similar outcomes in both conditions suggesting that either approach can be used based on one's preferences.

## Background

High-intensity interval training (HIIT) is a widely used training modality aimed to improve cardiorespiratory fitness among athletes in a range of sports, particularly endurance-based ones (e.g., runners, rowers, cyclists) [1, 2]. While HIIT can be prescribed in several ways, its basic tenets include repeated high-intensity and short-duration bouts (intervals) interspersed with rest periods [2, 3]. The improvement in VO_2_max following HIIT sessions is well documented [2, 4, 5], and presumed to result from the total time spent near VO_2_max (> 90% VO_2_max) [6–8]. Whereas the intervals increase oxygen consumption, the rest periods decrease oxygen consumption, which can lower the overall aerobic stimulus [9]. However, the rest periods allow subsequent intervals to be performed with sufficiently high intensity [9]. Accordingly, prescribing different rest durations in HIIT sessions could impact physiological and performance-related outcomes.

The rest duration in HIIT is traditionally prescribed using a fixed approach, in which athletes are prescribed active or passive rest periods of predetermined durations, with work-to-rest ratios ranging from 1:0.5 to 1:20 [2, 9]. While the fixed approach is straightforward, well-studied and effective, it has several shortcomings. First, a fixed rest duration does not account for inter- and intra-individual differences in physiological and performance outcomes. Some may prefer to rest for shorter durations between the first few intervals and finalize the session with longer rest durations; others may prefer the opposite. Accounting for one’s preferences via choice provision can lead to positive psychological effects [10–12] and, at times, better performance outcomes [13, 14] (although see examples of null effects [15, 16]). Finally, fixed rest duration does little to challenge athletes’ decision-making processes. Yet, in endurance competitions, athletes are required to strategically increase, decrease, or maintain speed as a function of the distance left to the finish line and their location relative to other competitors [2, 17]. Thus, incorporating self-selection into HIIT training could benefit both cardiovascular and strategic components.

An alternative approach for prescribing rest periods in HIIT is the self-selected (SS) approach, in which athletes choose the rest duration. The SS approach has the potential to account for the shortcomings of the fixed approach mentioned above. First, athletes choose the rest duration based on their current and anticipated performance, which can better account for individual differences. Second, the act of choosing can enhance motivation [11], enjoyment [12], and at times, motor performance [13, 14]. Third, the SS approach can challenge and improve athletes’ decision-making processes in competitions, by having them practice when and how to use their rest periods in training. Many studies have compared the fixed and SS approaches using different choice options (e.g., exercises’ order [18]) and different training modalities (e.g., resistance exercises [15]). Yet, only a handful of studies compared fixed and SS approaches using rest duration as the choice option during HIIT sessions, and these studies reported mixed results [19–25].

Some of the studies mentioned above resulted in subjects choosing shorter [20, 22] or longer [21, 23, 24] rest durations compared to the fixed conditions. Moreover, performance outcomes (e.g., covered distance or speed) varied between studies, resulting in superior [21, 23, 24] or inferior [20, 22] outcomes in the SS conditions. These inconsistent results may stem from several reasons, including the different HIIT protocols (e.g., 4 × 4 min [24] vs. 12 × 30 m [21]) and the rest durations provided in the fixed condition (e.g., 3 min [25] vs. 30 s [23]). Regardless, they all share a common study design feature—the total rest duration between conditions was not matched. That is, subjects selected their rest duration independently of the assigned rest duration in the fixed condition. This design precludes the ability to disentangle the direct effects of choice from the mediation caused by the total rest duration on performance, physiological, and psychological outcomes. Practically, uncontrolled rest durations allow subjects to select overly short or long rest durations, which can contradict the session’s goals. It can also prevent coaches from planning the overall session duration, given that the selected rest duration can vary considerably, especially in team settings. Finally, when the rest duration is unconstrained, the subjects’ decision-making process in each interval has little bearing on future intervals. In contrast, when the rest duration is close-ended, each choice concerning the rest duration has substantial effects on subsequent intervals. The latter can be presumed to have higher relevance to endurance competitions.

Accordingly, the present study aimed to compare, for the first time, the effects of fixed and SS conditions in a cycling HIIT protocol with an identical total rest duration. To this end, we performed a randomized cross-over study with 24 amateur male cyclists, measuring performance, physiological, and psychological outcomes. We chose cyclists as the study population due to their requirements for well-developed aerobic capacity and their common usage of HIIT [4, 26–28].

## Methods

### Subjects

We recruited 24 male amateur cyclists (mean (standard deviation); age: 36.6 (7.2) years; weight: 76.2 (12.7) kg; height: 1.75 (5.6) cm). Inclusion criteria included healthy cyclists between the ages of 18 and 45 who cycle for a total of at least 200 km a week for at least one year. We recruited cyclists through advertisements posted on various social media channels.

### Procedures

We implemented a randomized cross-over design. All cyclists attended three laboratory sessions: a familiarization session, and two experimental sessions, between three and eight days apart. In the familiarization session, after signing a consent form, we provided explanations about the protocol and outcomes, customized the seat height and handlebars of the SRM ergometer (Schoberer Rad Meßtechnik—SRM International, Jülich, Germany), and familiarized cyclists with the isokinetic mode of the SRM ergometer and the experimental conditions. The cyclists were block-randomized (50%–50% split) to first perform one of two conditions described below. Both sessions included a protocol composed of nine intervals lasting 30 s each, with the SRM ergometer limited to a maximal cadence of 90 revolutions per minute. Importantly, the cyclists were informed that the goal in both conditions was to maximize the total amount of watts produced over these nine intervals, as opposed to maximizing the watts output in every single interval. Under the fixed condition, cyclists rested for 90 s between intervals, totaling 720 s of rest (12 min). Under the SS condition, cyclists selected how long they would rest between the nine intervals. However, we matched the total rest duration between conditions, so that cyclists had to use 720 s of rest throughout the protocol (after the first and before the last interval) in any way they chose. Resting in both conditions consisted of active recovery of pedaling against a resistance of 50 watts. We inspected the test–retest reliability of the SS condition among ten cyclists who participated in a fourth session replicating the SS session.

The utilized protocol was decided upon due to two main reasons. First, we aimed to keep the work/rest ratio and the number of intervals within the range used in the HIIT literature [2, 9]. Second, we aimed to provide the cyclists with sufficiently long rest durations, so that the cardiovascular strain resulting from the completed intervals would minimally interfere with their decision-making abilities. The balance of the above-mentioned constraints was reached by conducting several pilot trials with different work/rest ratios.

**Fig. 1:**
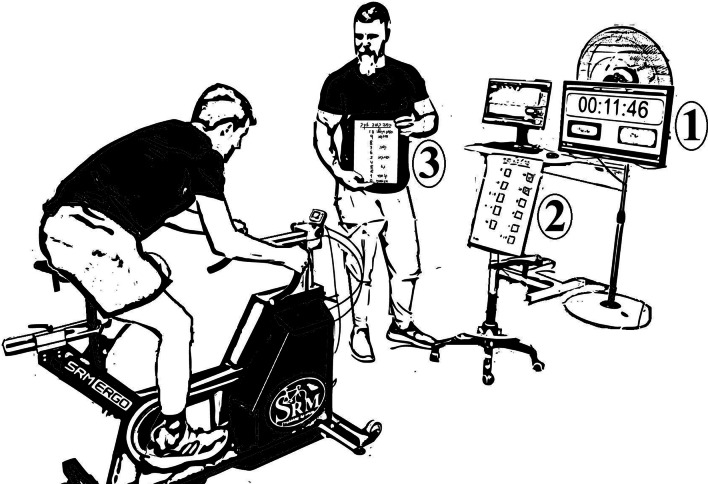
Experimental setup. (1) Computer screen with timer, (2) small whiteboard with list of completed intervals, and (3) clipboard with RPE and ROF scales

We presented cyclists with a timer on a computer screen next to the ergometer. In the fixed rest condition, the timer counted 90 s between intervals. In the SS condition, an on-screen 720-s countdown began during the rest periods (Fig. 1). When the cyclists announced they were ready to start the successive interval, the researcher began a 10-s verbal countdown, after which the interval would commence, and the timer paused. Once the set was completed, the countdown continued, and a checkmark was drawn on a board in front of the cyclist representing the completed sets. Cyclists completed the same incremental warmup in all sessions, consisting of three, four-minute bouts of cycling at a resistance of 100, 125, and 150 watts, respectively. After one minute of active rest, cyclists performed an all-out six-second sprint to assess their maximal power, followed by two minutes of active rest before performing the first set of the protocol. Two minutes after the last interval, cyclists completed another six-second maximal effort sprint to assess the impact of the protocol on maximal power, to provide an indication of fatigue. We recorded heart rate (HR) and electromyography (EMG) of the Vastus Lateralis (VL) and Biceps Femoris (BF) of their left leg. Cyclists also provided their rating of perceived effort (RPE) after each interval and their rating of perceived fatigue (ROF), perception of autonomy, and enjoyment after each session.

### Familiarization (Session 1)

To reduce the likelihood of potential biases, we told cyclists that the study’s goals were to examine the test–retest reliability of their watt production and heart rates over the sessions. Following the explanations, anthropometric measurements, and warmup, we familiarized participants with both experimental conditions. Cyclists completed a partial protocol composed of four intervals per condition. Specifically, cyclists performed four intervals under the fixed condition with 90 s of rest between each interval, for a total of 270 s (4.5 min). They then rested for five minutes and performed four intervals under the SS rest condition, in which they selected how long to rest between intervals, provided they used all 270 s. The order of the two conditions was randomized and counterbalanced between cyclists. The timer was reset every 90 s in the fixed condition or kept continuously running for 270 s in the SS condition during each rest period. Cyclists were instructed that the goal was to accumulate the maximum total amount of watts across intervals.

### Experimental Sessions (Sessions 2–3)

We briefly reviewed how to rate effort and fatigue using the different questionnaires, and had cyclists perform the warmup followed by the six-second sprint. After two minutes of active rest, cyclists completed the entire protocol, composed of nine 30-s intervals. The procedure was comparable to the familiarization session with two differences: cyclists only completed one of the conditions, and the protocol consisted of nine rather than four intervals. The latter implied that the total rest period between intervals was 720 s (90 s × 8 rest intervals). Hence, in the SS condition, the countdown began 720 s.

### Outcome Measures

#### Performance Measures

Watts: We measured watts produced on the SRM in the isokinetic mode at a rate of 1 Hz in each interval and the accumulated sum in each protocol.

### Physiological measures

HR: We measured the peak heart rate per minute of each interval using a complementary SRM chest strap monitor, which was stored on the same SRM recording software.

EMG: We measured muscle activity of the left leg VL and BF using two Tringo® Wireless Biofeedback System surface electromyography (EMG) sensors, attached and secured to the muscle bellies using customized glue strips and medical tape (Delsys Incorporated, Natick, MA, USA). The attachment location and position were determined using the SENIAM protocol (https://www.seniam.org). We used the power spectral density function to extract the median frequency (MDF) (See Additional file 1 for data preparation and preprocessing procedures).

### Psychological measures

RPE: After each interval, we presented the cyclists with a 0 (no effort) to 10 (maximal effort) RPE scale and asked them to provide an answer to the question, “How much effort did you exert?” with reference to the currently completed interval. In the familiarization session, cyclists received an explanation that effort is the process of investing mental and physical resources in a task, and that RPE is the perceived investment of one’s physical or mental resources to perform a specific task out of a perceived maximum [29]. Zero was anchored at total rest and 10 at cycling as fast and as hard as possible in a 30-s interval.

ROF: After completing the second sprint, we presented the cyclists with a 0 (not fatigued at all) to 10 (total fatigue and exhaustion—nothing left) ROF scale and asked them to provide an answer to the question, “How fatigued are you?”. We followed the recommendations by Micklewright et al. [30] on how to explain, instruct, and anchor the lower and upper values of the ROF scale.

Perceptions of autonomy: Approximately five minutes after the cool-down, we presented cyclists with three questions from the Intrinsic Motivation Inventory questionnaire [31, 32]: “1. The way I exercised today is aligned with my choices and preference.”, “2. I feel the way I exercised today is the way I want to exercise.”, and “3. I feel like I could make decisions regarding how I exercised today." to which the answers ranged from 1 (“I totally disagree”) to 5 (“I totally agree”).

Enjoyment: A few minutes after the cool-down, we presented cyclists with the question, “How much did you enjoy today’s session?” to which the answers ranged from 1 (“Not at all”) to 7 (“Exceptionally so”).

### Statistical Analysis

#### Single-Measurement Analyses

Paired t tests were used to derive confidence intervals (CI) and p values for the difference between the conditions for all outcome measurements (watts, HR, MDF VL and BF, and RPE, ROF, perception of autonomy and enjoyment). Note that outcomes measured multiple times across the session were aggregated by averaging over the intervals, thus providing a single number for each measurement, for each subject-condition combination.

#### Multiple-Measurement Analyses

When characterizing the changes of the different measurements over the intervals, we employed generalized additive mixed-effects models (GAMMs). GAMMs are an extension to generalized linear mixed-effects models, where the assumption of a linear effect of a covariate is replaced with a smooth estimation of said effect using splines [33]. We fitted GAMMs with a Gaussian distribution and identity link, thin-plate basis function, and a basis dimension of 3, with the following form:

1 $$y_{ijt} = \beta_{0 } + \gamma_{0i} + \beta_{{{\text{Cond}}}} \cdot j + f^{{{\text{rand}}}}_{ij} \left( t \right) + f^{{{\text{fixed}}}}_{j} \left( t \right) + \varepsilon_{ijt }$$

where $y$ is the measurement of interest (rest duration, watts, HR, MDF VL and BF, and RPE); *i* denotes each cyclist; *j* = *0,1* is the condition *(*fixed and self-selected, respectively); and *t* = *1,..,9* is the interval number (or the period between the sets, limited to *t* = *1,..,8*, when the dependent variable is the rest time). A random intercept was assigned to each subject ($\gamma_{0i}$), a random smooth term of *t* was assigned to each subject-condition combination ($f^{{{\text{rand}}}}_{ij} \left( t \right)$), and a fixed intercept ($\beta_{{{\text{Cond}}}}$) and a fixed smooth term of *set (*$f^{{{\text{fixed}}}}_{j} \left( t \right)$) were assigned to *condition.* When testing significance of the interaction between the smooth terms and *condition*, we changed the GAMMs formulation to increase statistical efficiency. In this case, a smoothed term was fitted to the interval number ($f^{{{\text{fixed}}}} \left( t \right)$) and another smooth term for any difference due to the self-selected group ($f^{{{\text{fixed}}}}_{{{\text{diff}}}} \left( t \right)$). The rest of the formulation was as in Eq. (1). Then, the p value for $f^{{{\text{fixed}}}}_{{{\text{diff}}}} \left( t \right)$ was estimated.

### Difference-in-Differences

When analyzing the pre–post six-second sprints, a difference-in-differences approach was used. That is, for each measurement, we subtracted each cyclist’s second sprint result from the first sprint result under the fixed condition, and subtracted it from the same difference under the self-selected condition. Similarly to the single-measurements, *t* tests were used to derive CIs and *p* values for this procedure.

### Test–Retest Analyses

All analyses of retests were performed analogously to the analyses described above, while using only ten cyclists who performed the retest (age: 35.6 (7.92) years; weight: 77.69 (12.99) kg; height: 1.76 (0.05) cm) and exchanging the fixed condition with a retest condition.

## Results

We present the study results stratified by outcome categories: rest duration, performance, physiological, and psychological outcomes. Note that due to the large number of outcome measures, only a subset is presented graphically, whereas summary and inferential statistics for all measured variables are presented in the tables in the main text and in the Additional file 1. Across all outcomes, the retest produced highly consistent results. Excluding the result duration outcome (presented below), the test–retest comparisons are presented in detail in the Additional file 1: (Tables S1, S4, S6, S8, S10, S12 and S13).

### Self-Selected Rest Duration

**Fig. 2:**
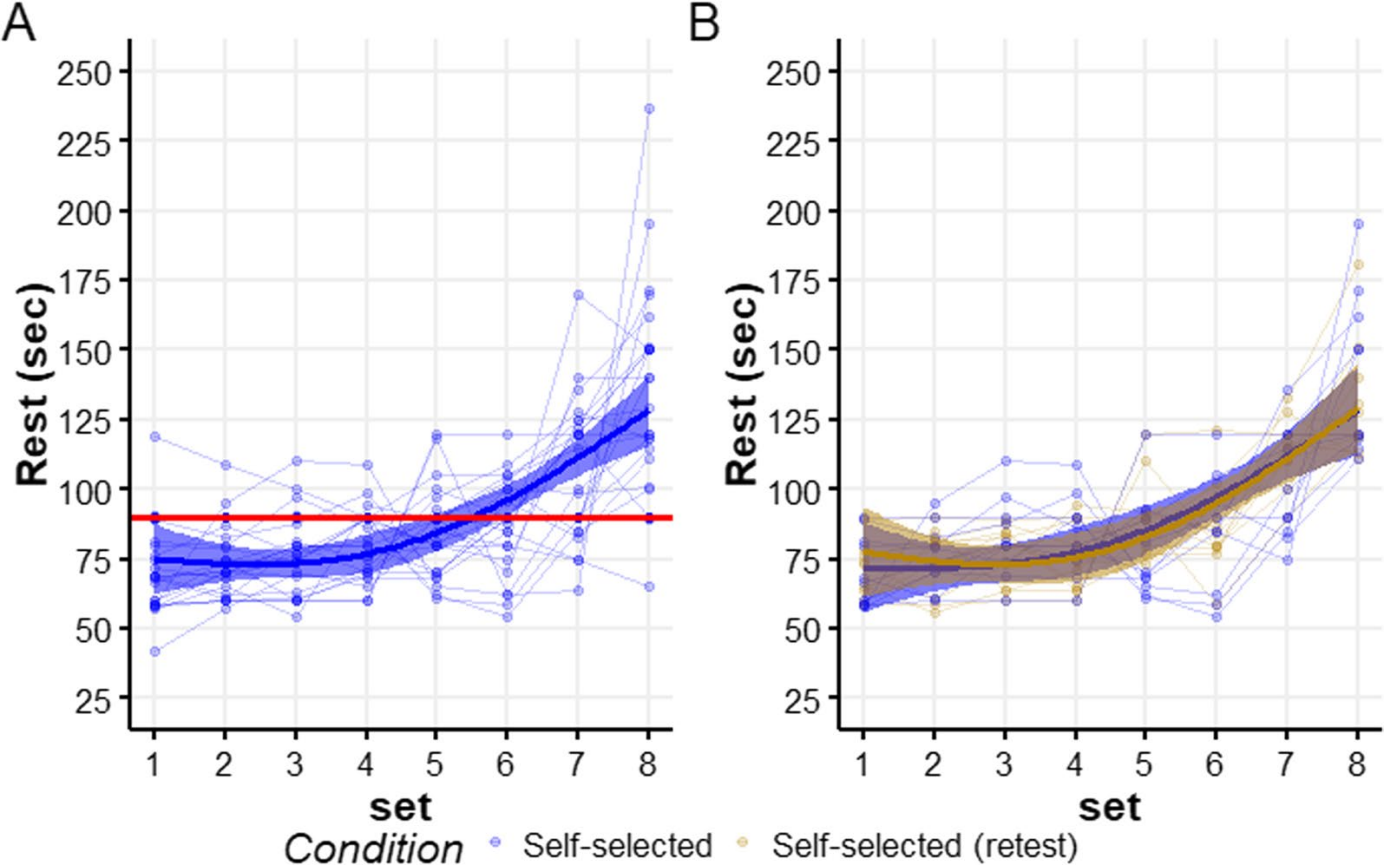
Self-selected rest durations between the nine intervals. The thin lines represent self-selected rest durations by the cyclists, whereas the thick lines and error bands represent a GAMM-based estimation of the average rest duration and their 95% CIs, respectively. **A** Comparison between the fixed and SS conditions for 24 cyclists. The red horizontal line represents the 90-s fixed rest duration allocated in the fixed condition. **B** Comparison between the test (blue) and retest (yellow) in a subsample of 10 cyclists, under the SS condition

We compared the SS rest duration to the 90 s rest allocated in the fixed condition. Between intervals 1–4, cyclists chose to rest for shorter durations; whereas, approximately after the sixth interval, cyclists considerably increased their rest durations (Fig. 2A). Although variability between cyclists’ rest durations was noticed, a very similar average pattern was observed in the retest of a subsample of ten cyclists (Fig. 2B; Additional file 1: Table S2).

### Performance Outcomes

**Fig. 3:**
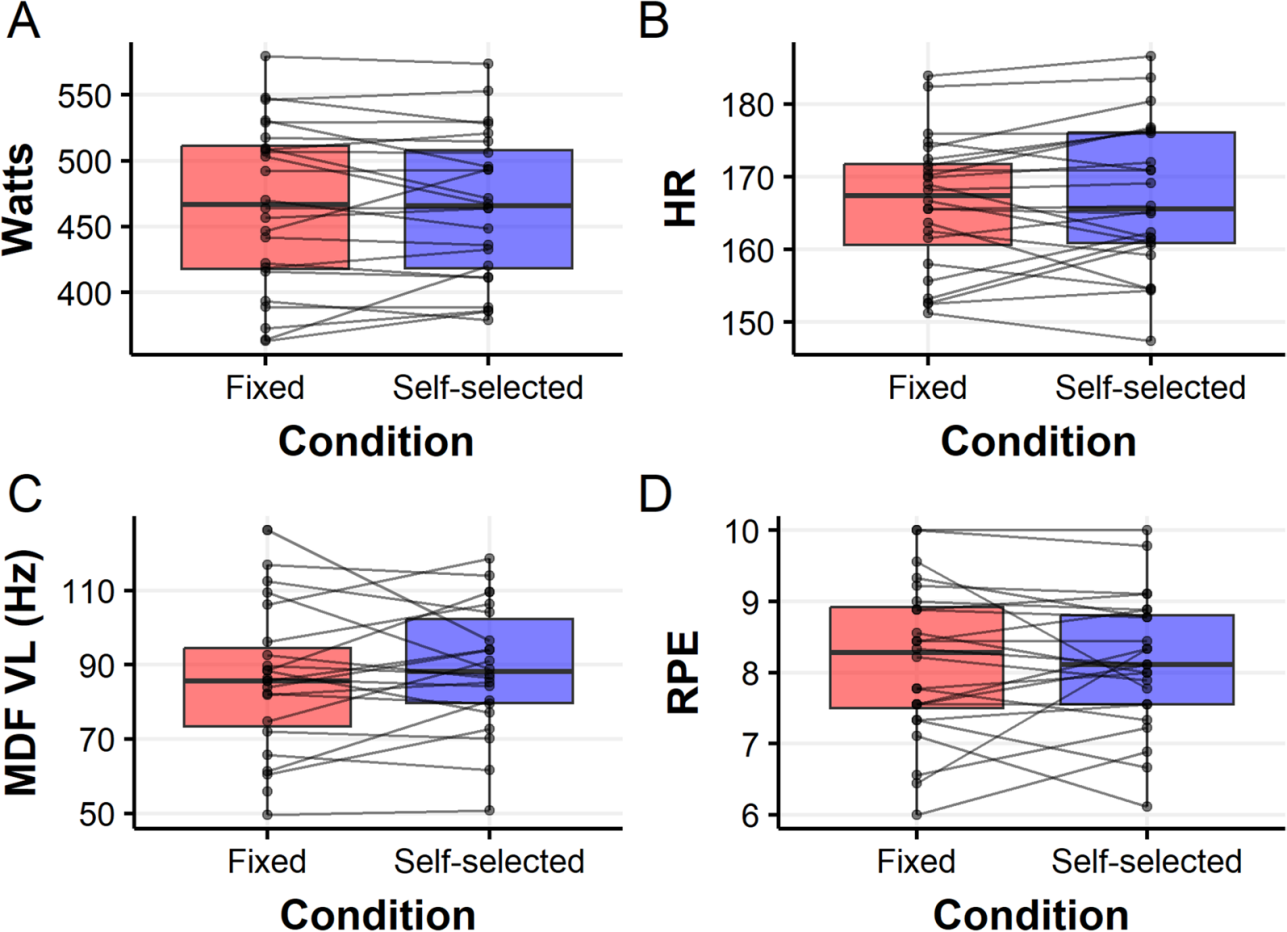
Comparisons of aggregated outcomes between fixed and SS conditions for Watts **A**, HR **B**, MDF VL **C**, and RPE **D**. Each horizontal line connects between the values of a single cyclist in both conditions, overlaid on boxplots

**Fig. 4:**
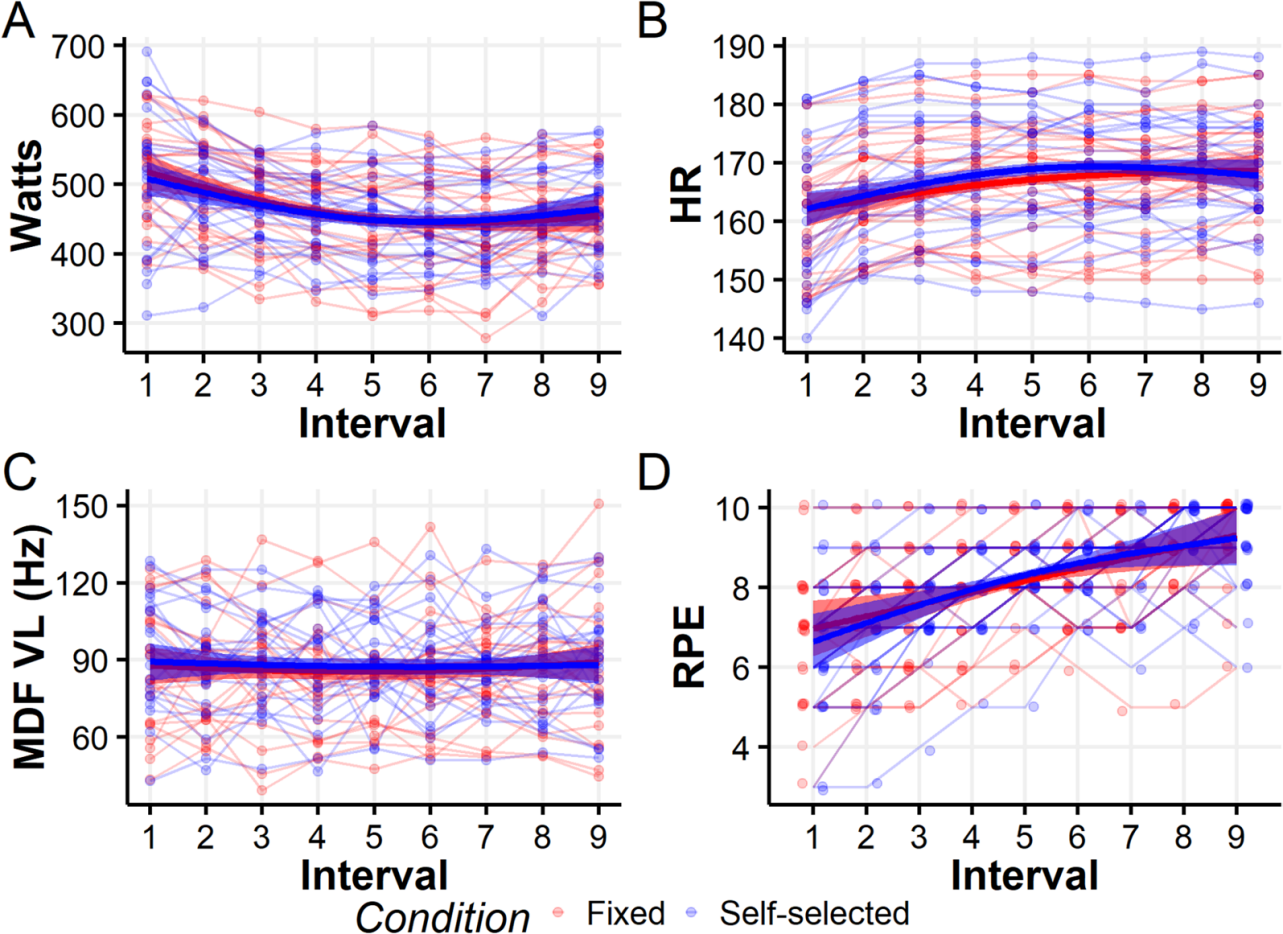
Multiple-measurement comparisons between fixed and SS in Watts **A**, HR **B**, MDF VL **C**, and RPE **D**. The thin lines represent individual outcomes of each cyclist, under the fixed (red) and SS (blue) conditions, whereas the thick lines and error bands represent a GAMM-based estimation of the average outcomes and their 95% CIs, respectively. Note that RPE measures were slightly jittered for clearer visualization, due to overlapping values (but left unchanged in the analyses)

Both the average watts throughout the sessions (Fig. 3A, Table 1) and patterns of watt production across the intervals (Fig. 4A, Additional file 1: Table S5) were highly similar under both conditions. We note the nonlinear patterns of watt productions: declining at first, plateauing mid-sessions, and slightly increasing toward the end. This end-sprint-like pattern was slightly more pronounced under the SS condition, as described above (Fig. 4A).

**Table 1:** Comparisons of single-measurements across intervals for self-selection and fixed conditions. *P* values and CIs derived from paired *t* tests

	Fixed (Means (SD))	Self-selected (Means (SD))	Mean difference (95%CI)	*P* value
Watts	466.18 (63.43)	465.60 (56.41)	0.57 (− 8.94, 10.09)	0.9
HR	166.34 (9.13)	167.19 (10.06)	− 0.85 (− 2.89, 1.18)	0.39
MDF VL	87.17 (19.63)	88.20 (16.92)	1.02 (− 4.75, 6.80)	0.71
MDF BF	67.9 (18.9)	62.2 (12.5)	5.60 (− 1.7, 13.1)	0.12
RPE	8.16 (1.09)	8.15 (0.94)	0.008 (− 0.29, 0.30)	0.95
ROF	7.66 (1.46)	7.62 (1.46)	− 0.04 (− 0.89, 0.81)	0.92
Autonomy 1	3.04 (1.37)	4.08 (1.18)	− 1.04 (− 1.88, − 0.20)	0.02
Autonomy 2	3.25 (1.11)	3.79 (1.10)	− 0.54 (− 1.20, 0.12)	0.1
Autonomy 3	2.58 (1.35)	4.17 (1.05)	− 1.58 (− 2.36, − 0.81)	0.0003
Enjoyment	5.50 (1.02)	5.50 (1.32)	0.00 (− 0.53, 0.53)	1

When inspecting the pre–post 6-seconds sprints, the patterns were again highly similar. The average watts produced in the pretest (Fixed: 913.7(170.0) watts; SS: 908.2(162.0) watts) and in the posttest (Fixed: 802.0(107.7) watts; SS: 808.7(110.9) watts), were similar, and produced a highly similar difference-of-differences (12.1, 95% CI (− 19.4,43.7); *p* value = 0.43).

### Physiological Outcomes

The average HR, MDF of the VL and BF throughout (Fig. 3B–C, Table 1) and across the session (Fig. 4B–C, Additional file 1: Tables S3, S7 and S9 accordingly) were highly similar under both conditions. We note the concave pattern of HR, in contrast to the linear, slightly decreasing pattern of VL.

### Psychological Outcomes

Four psychological outcomes were measured: RPE, ROF, autonomy, and enjoyment. RPE was the only outcome that was measured after every interval. Analogously to the outcomes described above, the average RPE throughout (Fig. 3D, Table 1) and across the session (Fig. 4D, Additional file 1: Table S11) were highly similar under both conditions. As might be expected, RPE increased with subsequent intervals, and this increase was approximately linear. ROF and enjoyment, which were measured at the end of each session, also exhibited highly similar results between the conditions (Table 1), indicating moderate-high levels of both perceived fatigue and enjoyment. Finally, perception of autonomy, which consists of three questions, was provided at the end of each session. The results of all three questions demonstrated a higher perceived autonomy under the SS condition, with differences ranging from about 0.5 to 1.5 mean estimates (on a scale of 1–7). However, the certainty around the mean estimates differed (Table 1).

## Discussion

We compared the effects of fixed and SS conditions in HIIT cycling sessions, while matching the total rest durations, on performance, physiological, and psychological outcomes, among 24 amateur male cyclists. Excluding perceptions of autonomy, which were higher in the SS condition, we found no statistical or substantial differences between the conditions in any other outcome. When inspecting the rest duration distribution, we found that cyclists selected to rest for shorter durations in the first five intervals, and for longer duration in the last three intervals, compared to the fixed condition. Furthermore, the same rest distribution was repeated in a retest session among the ten cyclists who completed it.

Although other studies have compared fixed and SS conditions in HIIT protocols [19–25], it is challenging to compare their findings to ours. This is because, to our knowledge, the present study is the only one that has matched the total rest duration between conditions. This specific study design has an important implication: it allows drawing conclusions regarding the direct effect of choice provision on the studied outcomes. Under a simple mediation model [34], the total effect of choice on outcomes can be broken down into (1) an indirect effect—the effect of choice mediated by rest (the effect that choice has on rest, which in turn has an effect on the outcomes); and (2) the direct effect—the effect that choice has on the outcomes, regardless of the effect of rest. This implies that when rest durations are not matched, it is impossible to tease apart the direct and indirect effects of choice. Due to the utilized experimental design, our study made some progress in uncovering the relationship between choice and performance, which seems to be almost fully mediated by the rest duration. We note that the effect of choice is still not fully identifiable in our study, because we were only able to match total rest duration, rather than rest duration per interval. That is, non-constant rest durations between intervals could have an effect regardless of choice, even when total rest is equated. The independent effect of choice can potentially be identified using a between-subject yoked control design, in which a subject in the fixed group (yoked) is imposed with a rest duration selected by a subject from the SS group (e.g., [35, 36]).

The practical implications of our results are that coaches and cyclists can choose to use either approach, based on their preferences or the qualities they aim to improve or monitor. Having the option to choose between the approaches can be beneficial, as each has its unique benefits. Using fixed rest durations provides cyclists with a degree of certainty regarding the session’s configuration, which may allow them to focus on power production and be less concerned with tactical decisions. Fixed rest durations in HIIT protocols can also be used to monitor changes in performance, both between and within cyclists, while minimizing variance due to non-physiological factors (e.g., tactical decisions). In contrast, using SS rest durations provides cyclists with certain flexibility regarding a session’s configuration. This can better account for between- and within-subject preferences and abilities. Some may prefer and benefit from shorter rest durations in the first few intervals, and longer rest durations in the latter intervals, whereas the opposite may be true for others. Over time, practicing such flexibility may place a greater focus on planning, exploring, and developing personalized rest allocation strategies. Finally, providing persons with choices is also known to have positive psychological effects [10–12].

To inspect the reliability of the SS condition, a subgroup of ten cyclists completed the SS session twice, and achieved similar results in all outcomes. Interestingly, the pattern of rest allocation selected by cyclists remained remarkably similar. The general pattern of selecting longer rest duration in later intervals is consistent with other SS rest duration studies [21, 25, 37] and has a sound physiological basis. When subjects are fresh in the first few intervals, they require less rest before initiating the next interval. In contrast, in later intervals, when fatigue has accumulated, longer rest durations are required to maintain or improve performance in subsequent intervals. The latter highlights a limitation of fixed and equal rest durations between intervals, as it may lead cyclists to rest for overly long durations initially, and for overly short durations in the final intervals.

This study has a number of limitations worthy of discussion. First, the HIIT protocol was conducted on an SRM ergometer using the isokinetic mode. While this allowed to exert control over cyclists’ cadence, and thus increase the degree of internal validity, it is unclear if the results generalize to more realistic cycling scenarios. Second, we only included male amateur cyclists. It is thus unclear if the observed results generalize to females, novice, and professional cyclists, or to other sports (e.g., running or rowing). Third, this was a short cross-over trial. It is thus unclear what long-term effects are to be expected under an SS training approach. Finally, we utilized a specific HIIT protocol. Other protocols may lead to different impacts on the rest allocation under the SS condition. For example, the allocated 12 min of rest duration in the SS condition may have been too long to allow for optimal decision-making with respect to goal of maximizing accumulation of watts. That is, deciding how to distribute this relatively long duration over eight rest periods may have been too challenging. The fact that cyclists were left with approximately two minutes of rest in the last rest period could stem from them inaccurately planning their rest allocation. However, the alternative, in which cyclists planned their rest allocation as part of a strategy is also likely. The latter is supported by the test–retest results in which cyclists chose the same rest allocation pattern. Future studies could examine if shorter SS rest durations, provided over fewer intervals, can lead to different results, and inspect when and why cyclists selected their rest allocation.

## Conclusion

To conclude, to our knowledge, this is the first study to compare the fixed and SS rest durations approaches on performance, physiological, and psychological outcomes, in a HIIT cycling protocol, with matched total rest durations. We observed highly similar outcomes under both conditions. These findings are practically useful, as they suggest that coaches and cyclists can follow either of the approaches based on their preferences and specific training goals.

## Supplementary Information

**Additional file 1.** EMG data preparation and preprocessing procedures.

## Data Availability

The raw data and Additional file 1 can be found online at https://osf.io/equwy

## Abbreviations

HIIT: High-intensity interval training
SS: Self-selected
CI: Confidence interval
HR: Heart rate
EMG: Electromyography
VL: Vastus lateralis
BF: Biceps femoris
RPE: Rating of perceived effort
ROF: Rating of perceived fatigue
SENIAM: Surface electromyography for the noninvasive assessment of muscles
MDF: Median frequency
GAMM: Generalized additive mixed-effects models
