# Supplementary material for: Should I Rest or Should I Go Now? A Randomized Cross-Over Trial Comparing Fixed and Self-Selected Rest Durations in High-Intensity Interval Training Cycling Sessions
Source: Sports Med Open. 2023 Jul 3;9:52. doi: 10.1186/s40798-023-00601-8 (PMC10317935; doi:10.1186/s40798-023-00601-8)
Supplement: Supplementary file 1 — Additional file 1. EMG data preparation and preprocessing procedures. [file 40798_2023_601_MOESM1_ESM.docx]

## Should I rest or should I go now? A randomized cross-over trail comparing fixed and self-selected rest durations in high-intensity interval training cycling sessions. Sports Med - Open

## Eyal Colorni^1, 2^, Evyatar Ohayon^2^, Julie N.Côté^3,4^, Uri Obolski^5,6,†^, Israel Halperin^1, 2,†*^

## ^1^ Department of Health Promotion, school of Public Health, Sackler Faculty of Medicine, Tel-Aviv

## University, Tel-Aviv, Israel

## ^2^ Sylvan Adams Sports Institute, Tel Aviv University, Tel-Aviv, Israel

## ^3^ Department of Kinesiology and Physical Education, McGill University, Montreal, Quebec, Canada

## ^4^ Occupational Biomechanics and Ergonomics Laboratory, Michael Feil and Ted Oberfeld/CRIR Research Centre, Jewish Rehabilitation Hospital, Laval, Quebec, Canada

## ^5^ Department of Epidemiology and Preventive Medicine, School of Public Health, Sackler Faculty of Medicine, Tel Aviv University, Tel Aviv, Israel

## ^6^ Department of Environmental Studies, Porter School of the Environment and Earth Sciences, Sackler Faculty of Exact Sciences, Tel Aviv University, Tel Aviv, Israel

^†^ Equal senior authors

## Corresponding author’s email: [ihalperin@tauex.tau.ac.il](mailto:ihalperin@tauex.tau.ac.il)

## **Supplementary information**

**EMG data preparation**

We first marked the specific area of each muscle group as recommended by SENIAM ([https://www.seniam.org](https://www.seniam.org/)). For the VL, we measured the distance between the anterior spina iliaca superior (ASIS) to the lateral side of the patella. We marked a point that was two thirds of this distance from the ASIS. For the BF, we measured the distance from the ischial tuberosity and the lateral epicondyle of the tibia and marked the middle point. We then shaved the skin around the target area and cleaned it with an isopropyl alcohol swab. After we dried the area with a gauze pad, we placed and secured the bipolar Delsys EMG sensors on the target areas using Delsys stickers and duct tape. The sensors’ output was recorded via EMGworks, with an auto filter of 20-450 Hz bandpass.

**EMG data preprocessing**

We processed the data using LabView version 2022 Q3 (National Instruments Corp., Austin, TX, USA) with a self-built algorithm and an sEMG sensor mounted on the SRM crank arm for tracking pedal cycles. The muscle activity was identified for each cycle from every set with a sampling rate of 1259Hz. The data was down-sampled to 1024Hz, filtered with Notch at 50Hz and Butterworth 4^th^ order bandpass of 15Hz to 500Hz. For each muscle, we chose a threshold that describes the same biomechanics position between each muscle burst to make sure repeatability exists. The power spectral density (PSD) function was applied to each selected portion with a Hanning window and no averaging. The PSD averaged [1], and the median frequency (MDF) were extracted [2]: Where j represents the motor unit’s coding rate in Hz and P is the power of each j at the frequency domain. The equation represents the definition of MDF as the integration of the total power (TTP) divided in two. A linear fit was applied to each set of MDF values, and the intercept was set to determine the value to describe the starting MDF of the set [1].

## **Supplementary References**

1. Bonato P, Roy SH, Knaflitz M, De Luca CJ. Time-frequency Parameters of the Surface Myoelectric Signal for Assessing Muscle Fatigue during Cyclic Dynamic Contractions. *IEEE Trans Biomed Eng.* 2001;48(7):745-753
2. Phinyomark, A. , Thongpanja, S. , Hu, H. , Phukpattaranont, P. , Limsakul, C. . The Usefulness of Mean and Median Frequencies in Electromyography Analysis. In: Naik, G. R., editor. *Computational Intelligence in Electromyography Analysis - A Perspective on Current Applications and Future Challenges* [Internet]. London: IntechOpen; 2012.

Table S1: Paired t-tests averaged across intervals for test-retest self-selection conditions

|  | **Self-selected 1** | **Self-Selected 2** | **Mean difference (95%CI)** | **P-value** |
| --- | --- | --- | --- | --- |
| **Watts** | 466.8 (59.54) | 461.08 (58.4) | 5.71 (-8.3 , 19.73) | 0.38 |
| **HR** | 168.84 (12.52) | 166.41 (11.75) | 2.43 (-1.3 , 6.17) | 0.17 |
| **MDF VL** | 92.9 (21.4) | 88.4 (17.0) | 4.5 (-6.8 , 15.9) | 0.38 |
| **MDF BF** | 56.0 (14.3) | 60.3 (14.9) | -4.3 (-10.0 , 1.4) | 0.12 |
| **RPE** | 8.09 (0.93) | 8.38 (0.88) | -0.29 (-0.9 , 0.32) | 0.31 |
| **ROF** | 7.66 (0.99) | 7.62 (1.46) | -0.04 (-0.62 , 1.17) | 0.99 |
| **Enjoyment** | 5.6 (0.84) | 5.5 (0.85) | 0.1 (-0.31 , 0.51) | 0.59 |

Table S2 - Test-retest rest durations

| **Coefficient** | **Estimate** | **SE** | **p-value** |
| --- | --- | --- | --- |
| $\beta_{0}$ | 89.72 | 2.2 | <0.001 |
| $\gamma_{0i}$ | - | - | 1.0 |
| $\beta_{Cond}$ | 0.12 | 3.11 | 0.968 |
| ${f^{rand}}_{ij}(t)$ | - | - | 0.958 |
| ${}f^{fixed}(t)$ | - | - | <0.001 |
| ${f^{fixed}}_{diff}(t)$ | - | - | 0.372 |

Table S3 - HR

| **Coefficient** | **Estimate** | **SE** | **p-value** |
| --- | --- | --- | --- |
| $\beta_{0}$ | 166.34 | 2.13 | <0.001 |
| $\gamma_{0i}$ | - | - | <0.001 |
| $\beta_{Cond}$ | 0.86 | 1.02 | 0.4 |
| ${f^{rand}}_{ij}(t)$ | - | - | <0.001 |
| ${}f^{fixed}(t)$ | - | - | <0.001 |
| ${f^{fixed}}_{diff}(t)$ | - | - | 0.042 |

Table S4 - Test-retest HR

| **Coefficient** | **Estimate** | **SE** | **p-value** |
| --- | --- | --- | --- |
| $\beta_{0}$ | 168.08 | 4.27 | <0.001 |
| $\gamma_{0i}$ | - | - | <0.001 |
| $\beta_{Cond}$ | 3.18 | 1.34 | 0.0194 |
| ${f^{rand}}_{ij}(t)$ | - | - | 0.0285 |
| ${}f^{fixed}(t)$ | - | - | <0.001 |
| ${f^{fixed}}_{diff}(t)$ | - | - | 0.2377 |

Table S5 - Watts

| **Coefficient** | **Estimate** | **SE** | **p-value** |
| --- | --- | --- | --- |
| $\beta_{0}$ | 466.18 | 14.97 | <0.001 |
| $\gamma_{0i}$ | - | - | <0.001 |
| $\beta_{Cond}$ | -0.57 | 8.65 | 0.947 |
| ${f^{rand}}_{ij}(t)$ | - | - | <0.001 |
| ${}f^{fixed}(t)$ | - | - | <0.001 |
| ${f^{fixed}}_{diff}(t)$ | - | - | 0.215 |

Table S6 - Test-retest Watts

| **Coefficient** | **Estimate** | **SE** | **p-value** |
| --- | --- | --- | --- |
| $\beta_{0}$ | 462.91 | 15.42 | <0.001 |
| $\gamma_{0i}$ | - | - | <0.001 |
| $\beta_{Cond}$ | 9.62 | 16.93 | 0.571 |
| ${f^{rand}}_{ij}(t)$ | - | - | <0.001 |
| ${}f^{fixed}(t)$ | - | - | <0.001 |
| ${f^{fixed}}_{diff}(t)$ | - | - | 0.309 |

Table S7 - MDF VL

| **Coefficient** | **Estimate** | **SE** | **p-value** |
| --- | --- | --- | --- |
| $\beta_{0}$ | 86.737 | 3.869 | <0.001 |
| $\gamma_{0i}$ | - | - | <0.001 |
| $\beta_{Cond}$ | 1.179 | 2.112 | 0.577 |
| ${f^{rand}}_{ij}(t)$ | - | - | 0.297 |
| ${}f^{fixed}(t)$ | - | - | 0.102 |
| ${f^{fixed}}_{diff}(t)$ | - | - | 0.564 |

Table S8 - Test-retest MDF VL

| **Coefficient** | **Estimate** | **SE** | **p-value** |
| --- | --- | --- | --- |
| $\beta_{0}$ | 89.048 | 6.018 | <0.001 |
| $\gamma_{0i}$ | - | - | <0.001 |
| $\beta_{Cond}$ | 4.330 | 3.794 | 0.256 |
| ${f^{rand}}_{ij}(t)$ | - | - | 0.408 |
| ${}f^{fixed}(t)$ | - | - | 0.523 |
| ${f^{fixed}}_{diff}(t)$ | - | - | 0.957 |

Table S9 - MDF BF

| **Coefficient** | **Estimate** | **SE** | **p-value** |
| --- | --- | --- | --- |
| $\beta_{0}$ | 67.531 | 3.236 | <0.001 |
| $\gamma_{0i}$ | - | - | <0.001 |
| $\beta_{Cond}$ | -5.703 | 2.442 | 0.0201 |
| ${f^{rand}}_{ij}(t)$ | - | - | 0.247 |
| ${}f^{fixed}(t)$ | - | - | 0.17 |
| ${f^{fixed}}_{diff}(t)$ | - | - | 0.936 |

Table S10 - Test-retest MDF BF

| **Coefficient** | **Estimate** | **SE** | **p-value** |
| --- | --- | --- | --- |
| $\beta_{0}$ | 61.303 | 4.647 | <0.001 |
| $\gamma_{0i}$ | - | - | <0.001 |
| $\beta_{Cond}$ | -4.406 | 2.37 | 0.065 |
| ${f^{rand}}_{ij}(t)$ | - | - | 0.2925 |
| ${}f^{fixed}(t)$ | - | - | 0.0821 |
| ${f^{fixed}}_{diff}(t)$ | - | - | 0.6409 |

Table S11 - RPE

| **Coefficient** | **Estimate** | **SE** | **p-value** |
| --- | --- | --- | --- |
| $\beta_{0}$ | 8.16 | 0.38 | <0.001 |
| $\gamma_{0i}$ | - | - | <0.001 |
| $\beta_{Cond}$ | -0.01 | 0.29 | 0.975 |
| ${f^{rand}}_{ij}(t)$ | - | - | <0.001 |
| ${}f^{fixed}(t)$ | - | - | <0.001 |
| ${f^{fixed}}_{diff}(t)$ | - | - | 0.123 |

Table S12 - Test-retest RPE

| **Coefficient** | **Estimate** | **SE** | **p-value** |
| --- | --- | --- | --- |
| $\beta_{0}$ | 8.45 | 0.66 | <0.001 |
| $\gamma_{0i}$ | - | - | <0.001 |
| $\beta_{Cond}$ | -0.2 | 0.54 | 0.702 |
| ${f^{rand}}_{ij}(t)$ | - | - | <0.001 |
| ${}f^{fixed}(t)$ | - | - | <0.001 |
| ${f^{fixed}}_{diff}(t)$ | - | - | 0.827 |

Table S13 - Paired t-test (diff-in-diff) sprints 1 and 2 for test-retest self-selected condition

|  | **Test** | | **Retest** | | **Mean difference of differences (95%CI)** | **P-value** |
| --- | --- | --- | --- | --- | --- | --- |
|  | **Pre**  **Mean (SD)** | **Post**  **Mean (SD)** | **Pre**  **Mean (SD)** | **Post**  **Mean (SD)** |  |  |
| **Watts** | 974.4 (214.4) | 818.8 (127.2) | 941.9 (193.6) | 786.9 (113.4) | 0.5 (-51.0, 52.0) | 0.98 |
